# Gastrointestinal Dysfunction Impact on Life Quality in a Cohort of Russian Patients with Parkinson's Disease I-III H&Y Stage

**DOI:** 10.1155/2022/1571801

**Published:** 2022-04-28

**Authors:** A. A. Pilipovich, O. V. Vorob'eva, S. A. Makarov, N. N. Shindryaeva, Yu D. Vorob'eva

**Affiliations:** ^1^Department of Nervous Disease, I. M. Sechenov First Moscow State Medical University (Sechenov University), Moscow, Russia; ^2^City Polyclinic No. 2 of the Moscow City Health Department, Moscow, Russia

## Abstract

**Background:**

There are still no clearly proven methods to slow down or stop the progression of Parkinson's disease (PD). Thus, improving the quality of life (QoL) of patients with PD becomes of primary importance. Autonomic dysfunction and its symptoms are known to worsen the quality of life in PD, but the degree of this influence is underinvestigated. Particularly, impacts of the separate significant gastrointestinal symptoms, such as dyspepsia, constipation, and abdominal pain, in PD should be more precisely evaluated with the help of specific scales.

**Objective:**

To assess the impacts of gastrointestinal dysfunction and its symptoms on PD patient's QoL using PDQ-39.

**Methods:**

111 PD patients in the I-III Hoehn and Yahr (H&Y) stage were enrolled in the study. The following scales were applied: UPDRS III, PDQ-39, GSRS, GDSS, MMSE, BDI, STAI-S, and STAI-T.

**Results:**

The linear regression model showed that the PDQ-39 SI depended on summary assessments GSRS-SI (*β* = 0.333, *p* < 0.001), BDI (*β* = 0.463, *p* < 0.001), and UPDRS III (*β* = 0.163, *p* < 0.05). The use of the stepwise method, adding GSRS-SI and UPDRS III scores to the BDI predictor, improved the model (R2 increased from 0.454 to 0.574). The investigation of GSRS domain's influence revealed that PDQ-39 SI had a significant correlation with almost all of them, but the regression analysis showed significant QoL impacts of only two factors: constipation and abdominal pain (*β* = 0.288, *p* < 0.01 and *β* = 0.243, *p* < 0.05 accordingly).

**Conclusions:**

Our results suggest a considerable negative influence of depression and gastrointestinal dysfunction (especially constipation and abdominal pain) on QoL of patients with PD. Their impact on QoL in patients with I-III H&Y stages of PD is more significant than that of motor symptoms. Therefore, the correction of depression and gastrointestinal dysfunction should be prioritized in PD therapy.

## 1. Introduction

Parkinson's disease (PD) is the second most common neurodegenerative disorder. It has a steadily progressive course and gradually leads to a permanent disability [1]. There are still no clearly proven methods to slow down or stop the progression of PD, which is why improving the quality of life (QoL) is practically the most valid way to help PD patients for the time being.

It is to be noted that subsequently QoL is regarded in the narrow sense as a “quality of health” and as an important PD outcome indicator for the management, care, and progression [2].

PD includes a wide spectrum of motor and nonmotor disorders, which can worsen the physical and psychological state of patients and the life of their family. Determining the most significant factor with negative influence on patients' QoL can help prioritize steps within the treatment plan.

The influence of different factors on the quality of life in PD was considered in numerous publications [3–5]. Many investigators had a concern on the negative influence of nonmotor disorders, such as depression, cognitive dysfunction, sleep disturbance, and certain autonomic disorders [6–10] on the QoL. Commonly, the evaluation of nonmotor disorders is performed using integral holistic scales such as NMSS, SCOPA, etc., which include a variety of different nonmotor symptoms, particularly some gastrointestinal ones. However, the spectrum of gastrointestinal disorders is wider, and they need to be qualitatively and quantitatively assessed, taking into account the severity of the symptoms. This is necessary for the optimum therapy selection that positively affects the general wellbeing and quality of the patient's life, as well as ensures an exceptional prognosis of the disease course.

Many of the autonomic PD symptoms are resistant to dopaminergic therapy and continue to progress despite the standard treatment. They require a separate specific therapy. Autonomic disorders often play a major role in advanced stages of PD and lead to severe complications (e.g., malnutrition, pulmonary aspiration, megacolon, intestinal obstruction, and even perforation). They can decrease the patients' QoL at any stage of PD including the earliest prodromic one [11, 12]. Dysfunction of the gastrointestinal tract (GIT) is considered as one of the most widespread (60–80% of patients) and earliest of the nonmotor PD manifestations [13, 14]. Some PD autonomic symptoms, such as constipation, are known to appear a decade earlier than motor symptoms. GIT dysfunction in PD is represented by a broad group of symptoms, such as weight loss, sialorrhea, dysphagia, symptoms of gastroparesis (including anorexia, early satiation, nausea, vomiting, gastric distention, and weight loss), constipation, intestinal dyskinesia, and small intestine bacteria overgrowth (SIBO) [15, 16]. The negative influence of gastrointestinal dysfunction and its symptoms on the total QoL was found at early stages of PD.

As mentioned above, the evaluation of PD gastrointestinal dysfunction is usually performed as a part of the integrative scale assessment of varieties of nonmotor disorders, which lead to difficulties in understanding of the specific symptom influence. Only isolated publications [17] used targeted gastrointestinal scales that could provide the detailed evaluation of single symptom severity influence on QoL. Such targeted analysis has never been performed in the Russian population.

This study aimed to estimate the impact of gastrointestinal dysfunction on the quality of life of patients with the PD I -III Hoehn and Yahr stages (assessed by PDQ-39).

We examined the following main hypotheses:

1. A negative association between the gastrointestinal dysfunction and QoL exists for PD patients
2. Motor impairments and emotional disturbances (anxiety and depression) are factors affecting gastrointestinal function and quality of life of PD patients
3. There are correlations between autonomic GIT dysfunctions assessing by GSRS and PDQ-39 scale domains (mobility, daily activity, emotional wellbeing, stigma, social support, cognition, communication, and bodily discomfort)
4. There is a correlation of the QoL (assessed by PDQ-39) and its single domains with GSRS domains (abdominal pain, reflux, diarrhea, indigestion, and constipation)

## 2. Methods

Subjects were recruited in the clinics of Sechenov University, Moscow, Russia. Approval was obtained from the Local Ethics Committee I.M. Sechenov First Moscow State Medical University of the Ministry of Health of Russian Federation (Sechenov University) (protocol No. 04–13, 10.apr.2013).

### 2.1. Inclusion Criteria

  Male or female patients ≥ 18 years of age
  Clinical diagnosis of idiopathic PD according to the UK Parkinson's Disease Society Brain Bank Diagnostic Criteria [18]
  the participant is willing and able to give informed consent for participation in the study

### 2.2. Exclusion Criteria

  Moderate or severe dementia (ICD-10, MMSE < 24)
  Psychiatric and other brain disorders
  Concomitant somatic or neurologic pathology diagnosed prior to the study
  Secondary Parkinson's disease

Motor symptom severity was assessed by the Unified Parkinson's Disease Rating Scale (UPDRS) part III; investigation was performed by the same neurologist during the “on” state of the patients. Scale maximum of 56 points indicates the most severe disturbances, and 0 (zero) indicates absence of any disturbances.

QoL was evaluated using the Parkinson's Disease Questionnaire (PDQ-39) [19]. PDQ-39 consists of 39 questions grouped into eight domains: mobility, daily activity, emotional wellbeing, stigma, social support, cognition, communication, and bodily discomfort; scores in individual domains are summed up to a total score. Each item is rated by a five-point scale: 0 = never, 1 = rarely, 2 = sometimes, 3 = frequently, and 4 = always/not possible at all. Lower scores reflect a better QoL [20]. Each individual total score is calculated as follows: 100x (the sum of the patient's scores in the 39 questions/4 × 39). The domain scores are calculated the same way. The total score for the PDQ-39 (Summary Index - PDQ-39 SI) varies from 0 (no disturbances) to 100 (severe disturbance). The PDQ-39 Summary Index (PDQ-39 SI) is a total score of the eight health domains. The gastrointestinal dysfunction was assessed using 2 scales: for gastrointestinal symptoms and dyspepsia.

### 2.3. Gastrointestinal Symptom Rating Scale (GSRS)

The gastrointestinal symptom rating scale (GSRS) is a reliable and validated questionnaire [21, 22] that utilizes a seven-level Likert scale (0 = not disturbed, 1 = slight discomfort, 2 = mild discomfort, 3 = moderate, 4 = relatively severe but tolerable, 5 = severe, and 6 = very severe discomfort). The score depends on intensity and frequency of symptoms experienced during the previous week. Fifteen questions are clustered into five domains: abdominal pain, reflux, diarrhea, indigestion, and constipation. All questions scores are summed up to a total score (from 0 to 90 points). High score indicates severe gastrointestinal symptoms [23]. Each individual total score is calculated as follows: 100x (the sum of the patient's scores of 15 questions /90).

The domain scores are calculated the same way. Each subscale was scored from 0 (least affected) to 100 (most affected), and GSRS Summary Index (GSRS- SI) is a total score of the five domains and calculated the same way as PDQ-39 SI.

### 2.4. Glasgow Dyspepsia Severity Score (GDSS)

The Glasgow dyspepsia severity score (GDSS) is a validated multidimensional disease-specific scale for dyspepsia [24]. The scale consists of 7 questions; the scores obtained from each question are summed up to a total GDSS score (from 0 to 20 points). The scale focuses on the following three aspects of dyspepsia: (1) frequency of dyspepsia symptoms and their effect on normal activity and ability to work; (2) need of physician examination and diagnostic investigations for dyspepsia; and (3) demand of over-the-counter and prescribed medication for dyspepsia. The total scale score was calculated by summation. A high score indicates severe dyspepsia [25]. To exclude PD patients with moderate or severe dementia, mini mental state examination (MMSE) and ICD-10 diagnostic criteria were applied. Characteristics of depression and anxiety were obtained through face-to-face interviews with the patients, the Beck Depression Inventory, and the State-Trait Anxiety Inventory.

### 2.5. Beck Depression Inventory

BDI by Beck et al., 1961, consists of 21 questions scored by a 4-point scale from 0 (symptoms absent) to 3 (severe symptoms), with a range of overall scores from 0 to 63. The total scale score was obtained by a simple summation. A high score (from 30 to 63) indicates severe depression; scores from 20 to 29 and from 10 to 19 indicate moderate and mild depression, respectively [26].

### 2.6. State-Trait Anxiety Inventory

STAI, by Spielberger et al.) consists of two subscales, each containing 20 items to assess reactive and personal anxiety levels. In the State subscale (STAI-S), respondents indicate how much each statement reflects “how they feel at this particular moment of time”. Each item is rated on a four-point Likert-type scale (0 = not at all, 1 = somewhat, 2 = moderately so, 3 = very much so). In the Trait subscale (STAI-T) respondents indicate how they generally feel on a four-point scale (0 = almost never, 1 = sometimes, 2 = often, 3 = almost always). Anxiety absent items are reverse score, and 20 items of each scale are then summed for total scores (up to 30 points - low level of anxiety; from 31 to 45 - moderate level anxiety; more than 45-high level anxiety) [25].

### 2.7. Statistical Analysis

A series of linear regression models applying the forced method were used in order to determine the gastrointestinal influence (assessed by GSRS and GDSS) on the QoL (scored on the PDQ-39 scale). A general linear model using forced and stepwise methods with age and gender control was used to investigate the complex effect of motor and emotional disturbances (anxiety and depression) on the PDQ-39 score. Correlations between PDQ-39 domains and GSRS domains applying Spearman's rank correlation coefficient have also been calculated. All statistical analysis was made using SPSS, version 17.

## 3. Results

111 PD patients (52 women and 59 men) were enrolled in the study. PD stages were defined according to the modified Hoehn and Yahr Rating Scale (H&Y). 3.6% of the enrolled patients had 1.0 stage of PD, 0.9% of them had 1.5 stage, 34.2% of the patients had 2.0 stage, and 27.9%, and 33.3% of the patients had 2.5 and 3.0 stages, respectively.

PD treatment included oral levodopa (56 patients), dopamine agonists (63 patients), amantadine (39 patients), and some patients were treatment-naive (24 patients). 89% of patients showed symptoms of gastrointestinal dysfunction: 53.5% abdominal pain, 49.5% reflux, 26.3% diarrhea, 70.7% indigestion, and 69.7% constipation. Symptoms of mild depression were observed in 46.5%, moderate in 12.1%, and severe in 1% of the PD patients; 40.4% of patients had no depression. A low level of state anxiety was observed in 7.6% of the patients, moderate in 50.9%, and severe in 49.1%. A low level of trait anxiety was observed in 3.2% patients, moderate in 35.5%, and severe in 64.5%. General characteristics of the patients are shown in Table 1.

Our first hypothesis, that gastrointestinal dysfunction is closely related to QoL of PD patients, was confirmed by significant positive correlation of GSRS-SI with PDQ-39 SI (Table 2). In contrast, no correlation between dyspepsia (assessed by the GDSS) and QoL was found.

Our investigation of the second hypothesis on the role of motor and emotional disturbances (anxiety and depression) on PD, gastrointestinal dysfunction, and QoL showed that the total score of QoL significantly correlated with the scores of all four scales: depression, anxiety STAI-S and STAI-T, and motor symptoms UPDRS III (Table 3), as opposed to GSRS-score gastrointestinal dysfunction, which correlated only with scores of BDI depression and STAI anxiety but did not correlate with UPDRS III (Table 3). Furthermore, the scales with significant correlations were assessed using regression analysis that was controlled for age and gender.

Received by the forced method, our total linear regression model for the QoL (scored according to PDQ-39 scale) showed that the most influential factors on the QoL were depression BDI (*β* = 0.463), gastrointestinal dysfunction GSRS-SI (*β* = 0.333), and motor symptoms UPDRS III (*β* = 0.163) (Table 4). Factors, like sex, age and anxiety scales indicators, had no significant influences on QoL (Table 4).

While building our total linear model by the stepwise including method, the addition of GSRS and UPDRS III scales to the BDI scale predictor improved the model (R2 changed from 0.454 to 0.574), but further addition of anxiety scales STAI had no positive influence on the model (Table 5).

The third hypothesis on association between gastrointestinal symptoms and domains of PDQ-39 was confirmed. Significant correlation between GSRS-SI and all PDQ-39 domains was observed. GDSS total score correlated significantly with mobility, emotional wellbeing, social support, cognition, bodily discomfort, but not with daily activity, stigma, and communication PDQ-39 domains (Table 6).

To test our fourth hypothesis, the correlation between PDQ-39 and the GSRS domains (abdominal pain, reflux, diarrhea, indigestion, and constipation) was calculated. PDQ-39 SI significantly correlated with most GSRS domains, except diarrhea (Table 7).

Studying the interconnections between single domains of PDQ-39 and GSRS, the following significant correlations were found (Table 7):

  Abdominal pain and constipation with all PDQ-39 domains
  Indigestion with all PDQ-39 domains except daily activity
  Reflux with emotional wellbeing, social support, cognition, communication, and bodily discomfort
  Diarrhea with emotional wellbeing, social support, cognition, communication, and bodily discomfort

General linear regression model showed that PDQ-39 SI significantly depends on 2 domains of GSRS, constipation and abdominal pain (Table 8). Selection by exception demonstrated that both values had approximately the same correlation with the quality of life, whereas the remaining 3 domains (reflux, diarrhea, and indigestion) showed no linear relationship with it (PDQ-39 SI).

## 4. Discussion

The objective of this study was to evaluate the impact of gastrointestinal dysfunction on early-stage PD patients' QoL (assessed by the PDQ-39). Mean PDQ-39 SI was 28.96 (SD 16.48) (Table 1). The most affected domains seemed to be stigma (37.10), emotional wellbeing (32.01), and bodily discomfort (31.68); but the social support domain was less affected (15.76).

The most comprehensive investigation in this field was done by Austrian authors Michal Lubomski et al. [17] Their evaluation of PDQ-39 SI demonstrated overall moderate QoL impairment in the PD cohort (29.2, SD 17.3), bodily discomfort domain was mostly affected (39.7, SD 26.8), followed by mobility (35.7, SD 28.7) and daily activity (32.2, SD 24.1), and the social support domain was affected the least (14.3, SD 19.3). Partial discrepancy among our and Austrian results may be explained by a difference in duration and stages of PD. Emotional aspects, such as stigma and emotional wellbeing, might be essential at the early stages (our cohort); but for more serious cases (Australian cohort) in advanced stages and with long duration of PD physical disability (bodily discomfort, mobility, and difficulties in daily activities) plays the leading role and demands substantial social support.

Earlier studies [14, 27] using nonmotor rating scales showed GIT symptoms prevalence up to 60% to 80% in PD patients and significance of GIT symptoms influence on the QoL. In our study, 89% of patients reported abnormal gastrointestinal functions. Total score of GIT dysfunction (GSRS) significantly correlated with the total score of QoL (PDQ-39) in our cohort. An analysis using a general linear regression model showed that gastrointestinal dysfunction negatively influenced QoL, and a high score on GSRS should be considered a predictor for poor QoL (Table 2).

According to several studies, it was observed that affective disorders, particularly depression [28], may have a direct negative influence on the QoL of PD patients [29, 30]. In this research [31], a scale for nonmotor PD symptoms (NMS) was used, and relative impact of each clinical subscale on QoL was assessed. According to this study, the influence of depression was two times more significant than that of motor symptoms on health state. Anxiety and other nonmotor symptoms were shown to be important determinants of poor health state and QoL in PD.

Our study also demonstrated a link between patients' QoL and affective and motor symptoms. The affective status evaluation showed a broad distribution of anxiety-depressive symptoms in our patients. Mild/moderate depression was observed in 59.6% of patients, and more than half of them had evident anxiety. According to our data, anxiety and depression disorders had a significant correlation with QoL and GIT symptoms, and less correlation with motor symptoms (Table 3). Regression analysis with general linear modeling showed a significant and approximately equal contribution of depression and GIT dysfunction to the QoL, and less but also significant contribution of motor disorders on the QoL (Table 4). These three factors can serve as predictors for a patient's QoL. Our data showed that adding anxiety score to the model did not change the model accuracy (Table 5). We have shown a significant negative influence of GIT dysfunction on PD patient QoL, and this influence is comparable to that of depression and motor disorders.

Detailed studying of the GIT dysfunction revealed its influence on all aspects of QoL: mobility, daily activities, emotional wellbeing, stigma, social support, cognition, communication, and bodily discomfort of PD patients (Table 6). Dyspepsia severity according to GDSS also had correlation with several components of QoL such as mobility, emotional wellbeing, social support, cognition, and bodily discomfort (Table 6). However, the GDSS total score scale had no predictive value on the QoL (Table 2).

Among the GIT dysfunction symptoms found in our patients, the most frequent ones were dyspepsia (70.7%) and constipation (69.7%). Abdominal pain of different extent was present in approximately half of our patients (53.5%), and reflux symptoms - in 49.5%. Diarrhea was the rarest symptom (26.3%) (Table 1). These results agree with the data of earlier studies [32] that showed a high frequency of GIT dysfunction, especially constipation (50–80%), in PD patients. Kenna JE et al. found that “people with PD reported a greater frequency of constipation and GIT-associated disturbances when compared to healthy controls; total GSRS scores, upper GIT symptoms and hypoactive GIT symptoms were all significantly more prevalent in the PD cohort than controls” [33].

According to our data, all GIT dysfunction symptoms, except diarrhea, significantly correlated with the total score of PDQ-39 (Table 7). However, only abdominal pain and constipation had a significant impact (Table 8). These two symptoms significantly influenced all domains of a patients' quality of life measured with PDQ-39 (Table 7). Similar results were obtained in the Chinese research [34], where constipation had a relatively higher prevalence in patients with PD, PD patients with constipation had a higher incidence of depression, leading to worsening of the quality of life. Today, the link between the constipation and depression is well known in both patients with PD plus depression [35, 36] and in patients with major depression without PD [37]; this may explain increased importance of constipation influence on QoL in comparison with other GIT symptoms.

Correction of GIT and depression symptoms should be considered as an important part of therapy in early stages of PD. Revealing GIT dysfunction in early PD will lead to more effective therapeutic intervention, will help to avoid a series of complications and vastly improve patient QoL. Further research is needed to develop a strategy for controlling the factors influencing the QoL of PD patients, which requires a multidisciplinary approach.

Our study has some limitations. First, the patients were not examined by a gastroenterologist, nor any gastroenterological investigations were performed. The study was based on questionnaires filled by patients independently. Second, only PD outpatients, i.e., PD patients with mild to moderate stages, were included in our study. Additionally, patients with moderate to severe dementia were excluded. Therefore, it would be more appropriate to generalize the data from our study to patients with mild to moderate stage PD without severe dementia who applied to outpatient clinics.

Despite these limitations, our study is the first study to assess the impact of gastrointestinal dysfunction on QoL among Russian patients with PD, by using validated and reliable GSRS scoring.

## 5. Conclusion

Our results have indicated that gastrointestinal dysfunction (especially constipation and abdominal pain) as well as depression have a negative influence on the QoL of patients in early PD stages. Their impact on the I-III H&Y stage of PD is more significant than the impact of motor symptoms.

## Figures and Tables

**Table 1:** Demographic and baseline clinical characteristics of the patients.

Data	Mean (%)	SD	Range
Age (years)	63.33	8.265	42–80
PD duration (years)	4.65	2.58	1–13
Levodopa (mg)	215.41	254.474	0–1000
Stage H&Y (1–5)	2.5 (2, 3)^*∗*^	—	1–3
UPDRS-III (on state) (0–56)	28.51	10.7	8–64
PDQ-39SI (total score 0–156)	28.96 (45.17)	16.48 (25.723)	2.56–83.97 (4–131)
PDQ-39 mobility (0–40 score)	29.90 (11.94)	22.48 (8.993)	0–95 (0–38)
PDQ-39 daily activity (0–24 score)	31.3 (7.45)	23.80 (5.713)	0–100 (0–24)
PDQ-39 emotional wellbeing (0–24 score)	32.01 (7.72)	21.37 (5.129)	0–100 (0–24)
PDQ-39 stigma (0–16 score)	37.10 (5.99)	27.45 (4.393)	0–100 (0–16)
PDQ-39 social support (0–12 score)	15.76 (1.90)	19.60 (2.352)	0–75 (0–9)
PDQ-39 cognition (0–16 score)	26.35 (4.20)	19.09 (3.055)	0–100 (0–16)
PDQ-39 communication (0–12 score)	18.16 (2.17)	20.26 (2.432)	0–83.33 (0–10)
PDQ-39 bodily discomfort (0–12 score)	31.68 (3.82)	22.77 (2.733)	0–83.33 (0–10)
GSRS-SI (total score 0–90)	12.27 (11.05)	11.43 (10.287)	0–48.89 (0–44)
GSRS abdominal pain (0–12 score)	10.43 (1.25)	14.20 (1.704)	0–66.67 (0–8)
GSRS reflux (0–18 score)	8.13 (1.46)	10.62 (1.913)	0–38.89 (0–7)
GSRS diarrhea (0–18 score)	4.32 (0.78)	10.24 (1.844)	0–66.67 (0–12)
GSRS indigestion (0–24 score)	14.98 (3.60)	19.06 (4.576)	0–83.33 (0–20)
GSRS constipation (0–18 score)	21.94 (3.95)	23.94 (4.310)	0–100 (0–18)
GDSS (0–20 score)	3.37	3.625	0–19
MMSE (0–30 score)	28.29	1.562	24–30
BDI (0–63 score)	11.81	7.470	0–33
STAI-S	44.18	11.548	20–75
STAI-T	48.35	10.101	20–90

SD: standard deviation; H&Y: Hoehn and Yahr Rating Scale; UPDRS-III: The Unified Parkinson's Disease Rating Scale part III; PDQ-39: the Parkinson's Disease Questionnaire; SI: summary index; GSRS: gastrointestinal symptom rating scale; GDSS: Glasgow Dyspepsia Severity Score; MMSE: Mini Mental State Examination; BDI: Beck Depression Inventory; STAI-S: state subscale of the State-Trait Anxiety Inventory; STAI-T: trait subscale of the State-Trait Anxiety Inventory. ^*∗*^Me (Q1; Q3).

**Table 2:** Generalized linear model coefficients for PDQ-39 SI on both gastrointestinal assessments (GSRS and GDSS).

Model dependent variable: PDQ-39 SI		Unstandardized coefficients		Standardized coefficient	t	*p*	95.0% CI for B	
		B	SE	*β*			Lower	Upper
1	Constant	16.044	2.012		7.975	**≤0.001**	12.048	20.040
	GSRS-SI	0.708	0.124	0.522	5.701	**≤0.001**	0.462	0.955
	GDSS	0.779	0.400	0.178	1.947	0.055	−0.016	1.575

Bold indicates values of the regression equation for predicting the dependent variable from the independent variable; SE: standard error; *β*: beta; *t*: *t*-value; *p*: 2-tailed *p* value (significance level); CI: confidence interval.

**Table 3:** Spearman correlations (pairwise) of BDI, STAI-S, STAI-T, and UPDRS III with GSRS SI and PDQ-39 SI total scores.

Spearman coefficient	BDI	STAI-S	STAI-T	UPDRS III
PDQS-39 SI	0.666^*∗∗∗*^	0.422^*∗∗∗*^	0.449^*∗∗∗*^	0.223^*∗*^
GSRS-SI	0.564^*∗∗∗*^	0.291^*∗∗*^	0.430^*∗∗∗*^	0.062

^*∗*^ *p* < 0.05; ^*∗∗*^*p* < 0.01; ^*∗∗∗*^*p* < 0.001.

**Table 4:** Regression coefficients in the linear model for the quality of life PDQ-39 with scores according to gastrointestinal GSRS, motor symptoms UPDRS III, depression BDI, and anxiety STAI scales.

Model dependent variable: PDQ-39 SI		Nonstandardized coefficients		Standardized coefficient	t	*p*	95.0% CI	Correlation	
		B	SE	*β*			Lower	Upper	Zeroth order
1	Constant	−2.787	12.058		−0.231	0.818	−26.807	21.234	
	BDI	0.973	0.219	**0.463**	4.445	**0.000**	0.537	1.410	**0.674**
	GSRS-SI	0.451	0.121	**0.333**	3.719	**0.000**	0.209	0.692	**0.614**
	UPDRS III	0.285	0.135	0.163	2.117	**0.038**	0.017	0.553	0.237
	STAI-S	0.057	0.133	0.042	0.433	0.666	−0.207	0.321	0.400
	STAI-T	0.024	0.161	0.015	0.149	0.882	-0.297	0.345	0.386
	Age	0.022	0.155	0.011	0.140	0.889	−0.288	0.331	0.211
	Sex	0.673	2.607	0.021	0.258	0.797	−4.521	5.866	−0.153

Bold indicates values for regression equation predicting the dependent variable according to the independent variable; SE: standard error; *β*: beta; *t*: *t*-value; *p*: 2-tailed *p* value (significance level); CI: confidence interval.

**Table 5:** Summary results of generalized linear models (received by stepwise including method) of GIT dysfunction, motor disturbances, depression, and anxiety influences on QoL.

Model	R	R^2^	Adjusted R^2^	SE of the Estimate
0	0.674	0.454	0.448	11.815
1	0.740	0.547	0.536	10.833
2	0.758	0.574	0.558	10.567

Model 0 includes BDI; model 1 contains Model 0 plus GSRS- SI; model 2 includes Model 1 plus UPDRS III; PDQ-39 SI is a dependent variable. R: the square root of R2; R2: variance portion in the dependent variable, which can be predicted using the independent variables; SE: standard error.

**Table 6:** Spearman correlations (pairwise) of selected PDQ-39 domains with both gastrointestinal scales assessments.

PDQ-39	Mobility	Daily activity	Emotional wellbeing	Stigma	Social support	Cognition	Communication	Bodily discomfort
GSRS-SI	**0.406** ^*∗∗∗*^	**0.253** ^*∗*^	**0.452** ^*∗∗∗*^	**0.269** ^*∗∗*^	**0.408** ^*∗∗∗*^	**0.493** ^*∗∗∗*^	**0.382** ^*∗∗∗*^	**0.484** ^*∗∗∗*^
GDSS	**0.364** ^*∗∗∗*^	0.116	**0.340** ^*∗∗*^	0.091	**0.303** ^*∗∗*^	**0.235** ^*∗*^	0.180	**0.298** ^*∗∗*^

^*∗*^ *p* < 0.05; ^*∗∗*^*p* < 0.01; ^*∗∗∗*^*p* < 0.001.

**Table 7:** Spearman correlations (pairwise) of PDQ-39 scale and its domains with GSRS domains.

GSRS PDQ-39	Abdominal pain	Reflux	Diarrhea	Indigestion	Constipation
PDQ-39 SI	**0.498** ^*∗∗∗*^	**0.324** ^*∗∗*^	0.141	**0.399** ^*∗∗∗*^	**0.442** ^*∗∗∗*^
Mobility	**0.295** ^*∗∗*^	0.195	0.056	**0.304** ^*∗∗*^	**0.370** ^*∗∗∗*^
Daily activity	**0.245** ^*∗*^	0.112	−0.082	0.170	**0.259** ^*∗*^
Emotional wellbeing	**0.317** ^*∗∗*^	**0.294** ^*∗∗*^	**0.253** ^*∗*^	**0.386** ^*∗∗∗*^	**0.355** ^*∗∗∗*^
Stigma	**0.254** ^*∗∗*^	0.166	−0.78	**0.217** ^*∗*^	**0.288** ^*∗∗*^
Social support	**0.401** ^*∗∗∗*^	**0.281** ^*∗∗*^	**0.212** ^*∗*^	**0.233** ^*∗*^	**0.355** ^*∗∗∗*^
Cognitions	**0.497** ^*∗∗∗*^	**0.324** ^*∗∗*^	**0.300** ^*∗∗*^	**0.383** ^*∗∗∗*^	**0.368** ^*∗∗∗*^
Communication	**0.397** ^*∗∗∗*^	**0.227** ^*∗*^	0.172	**0.319** ^*∗∗*^	**0.299** ^*∗∗*^
Bodily discomfort	**0.389** ^*∗∗∗*^	**0.452** ^*∗∗∗*^	**0.198** ^*∗*^	**0.391** ^*∗∗∗*^	**0.316** ^*∗∗*^

^*∗*^ *p* < 0.05; ^*∗∗*^*p* < 0.01; ^*∗∗∗*^*p* ≤ 0.001.

**Table 8:** Regression coefficients of the general linear model for the life quality dependence PDQ-39 on GSRS domain scores.

Model dependent variable: PDQ-39 SI		Unstandardized coefficients		Standardized coefficient	T	*p*	95.0% CI	
		B	SE	*β*			Lower	Upper
1	Constant	19.136	2.248		8.514	**≤0.001**	14.672	23.599
	GSRS abdominal pain	0.294	0.140	0.243	2.107	**0.038**	0.017	0.572
	GSRS reflux	0.050	0.163	0.031	0.308	0.759	-0.273	0.373
	GSRS diarrhea	0.131	0.150	0.078	0.873	0.385	-0.167	0.428
	GSRS indigestion	0.083	0.103	0.092	0.804	0.424	-0.122	0.287
	GSRS constipation	0.207	0.077	0.288	2.688	**0.009**	0.054	0.359

Bold indicates values for regression equation predicting the dependent variable from the independent variable; SE: standard error; *β*: beta; *t*: *t*-value; *p*: 2-tailed *p* value (significance level); CI: confidence interval.

## Data Availability

The data used to support the findings of this study are available from the corresponding author upon request.
